# A Case of a Laryngeal MALT Lymphoma in a Patient with a History of Gastric MALT

**DOI:** 10.1155/2015/109561

**Published:** 2015-01-18

**Authors:** Mark Ashamalla, Marita S. Teng, Joshua Brody, Elizabeth Demicco, Rahul Parikh, Kavita Dharmarajan, Richard L. Bakst

**Affiliations:** ^1^Sidney Kimmel Medical College at Thomas Jefferson University, Philadelphia, PA 19107, USA; ^2^Department of Otolaryngology, Icahn School of Medicine at Mount Sinai Hospital, New York, NY 10029, USA; ^3^Department of Medical Oncology, Icahn School of Medicine at Mount Sinai Hospital, New York, NY 10029, USA; ^4^Department of Pathology, Icahn School of Medicine at Mount Sinai Hospital, New York, NY 10029, USA; ^5^Department of Radiation Oncology, Mount Sinai St. Luke's Roosevelt and Mount Sinai Beth Israel, New York, NY 10029, USA; ^6^Department of Radiation Oncology, Icahn School of Medicine at Mount Sinai Hospital, New York, NY 10029, USA

## Abstract

We are reporting a case of a 62-year-old African American woman with a history of gastric MALT lymphoma successfully treated with radiation who presented with a laryngeal MALT lymphoma 4 years after her original diagnosis. She received definitive radiation with a complete response. The case presented is unique for the rare presentation of a MALT lymphoma in the larynx, especially in light of the patient's previously treated gastric MALT lymphoma years ago.

## 1. Introduction

Mucosa associated lymphoid tissue lymphomas, or MALT, are extranodal marginal zone B-cell lymphomas. While the most common anatomical location is the stomach, MALT lymphomas can also present in the lung, thyroid, breast, synovium, lacrimal and salivary glands, orbit, dura, skin, and soft tissues [1–13].

Laryngeal MALT lymphomas are exceedingly rare. Our search of the current literature revealed only 27 cases reported between 1990 and 2014 [14–16]. Caletti et al. previously reported a case of a patient with concomitant laryngeal MALT lymphoma and* Helicobacter pylori* (*H. pylori*) related gastric MALT lymphoma [17], but to our knowledge, a representation of a MALT lymphoma in the larynx following a gastric MALT lymphoma has never previously been described. Given the rarity of laryngeal MALT lymphomas, the risk factors, prognosis, and optimal management are not well defined.

## 2. A Case Report

In June 2010, a 62-year-old female presented to her gastroenterologist with intermittent epigastric pain for several months. Subsequent endoscopy revealed a gastric lesion. Immunohistochemistry was strongly positive for CD20 and negative for CD5, CD10, and BCL-6, confirming the diagnosis of MALT lymphoma. Testing was negative for* H. pylori*. Imaging with a PET/CT confirmed a malignancy confined to the stomach with no lymph node avidity or other evidence of disease. t(11;18) status was not performed at the time.

**Figure 1:**
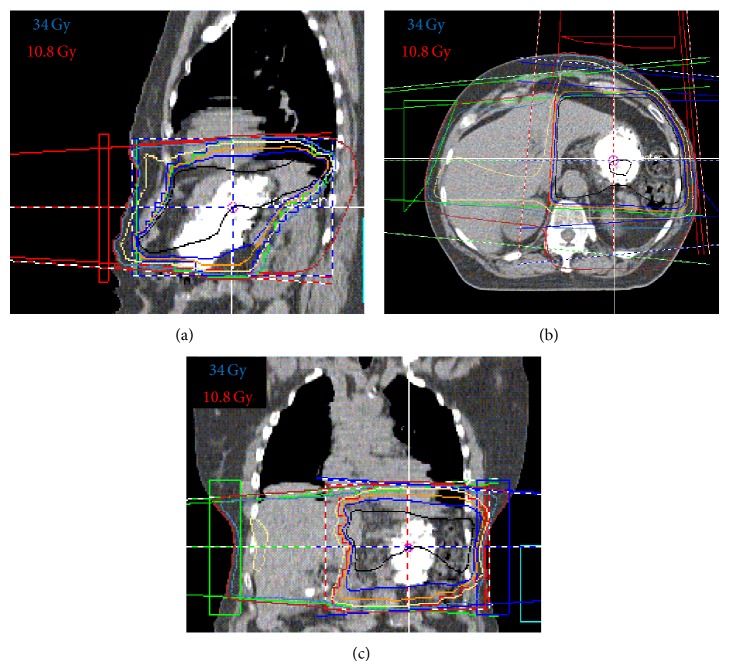
3D conformal radiation therapy of MALT lymphoma of the stomach. Dose distribution in the saggital (a), axial (b), and coronal (c) planes. Black line represents the 36 Gy isodose line.

The patient received definitive radiation to her stomach, upper abdomen, and perigastric lymph nodes to a total dose of 3600 cGy at 180 cGy per fraction in 20 fractions over 3 weeks (Figure 1). The patient tolerated treatment well. The patient was followed with serial endoscopies and remained without evidence of disease.

Approximately 4 years following her initial diagnosis, she presented to her gastroenterologist complaining of one year of progressive difficulty breathing, dysphagia, odynophagia, and right ear fullness. Subsequent workup with esophagogastroduodenoscopy (EGD) revealed an exophytic, polypoid mass in the right false cord and pyriform sinus.

**Figure 2:**
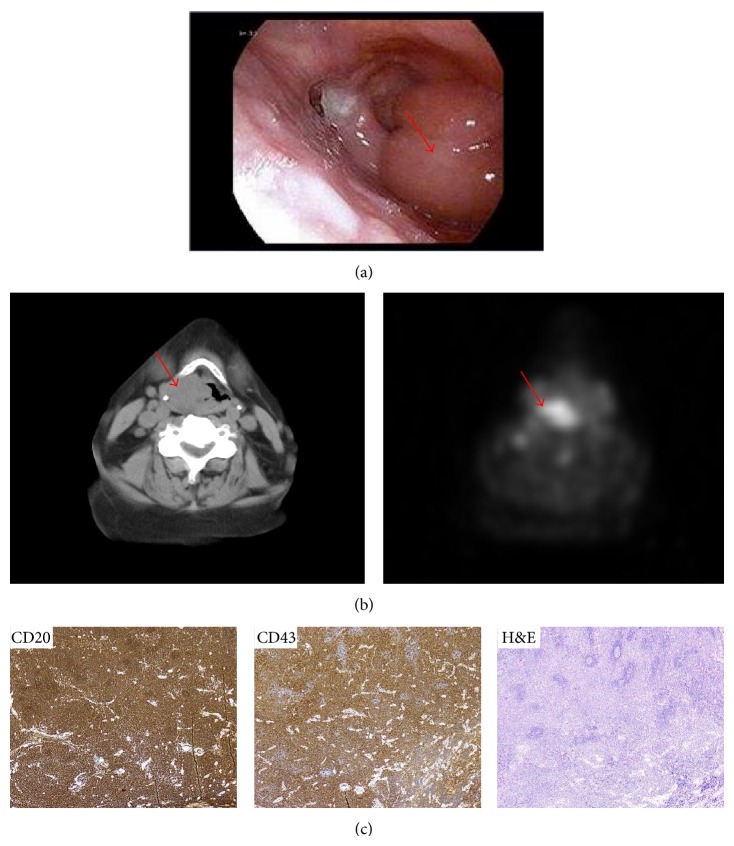
Laryngeal MALT lymphoma. (a) A 3 cm mass arising from the right aryepiglottic fold obscuring view of the right vocal cord on fiberoptic examination (red arrow). (b) PET/CT demonstrates a PET-avid exophytic mass arising from the right false cord (red arrow). (c) Immunohistochemistry: high power view (40x) anti-CD20, anti-CD43, and H&E: dense lymphocytic infiltrate and scattered small reactive-appearing follicles are seen surrounded by diffuse sheets of neoplastic marginal zone cells.

A fiberoptic examination confirmed a 3 cm mass arising from the right aryepiglottic fold obscuring view of the right vocal cord (Figure 2(a)). The left vocal cord was minimally visible upon phonation while the vallecula, epiglottis, and pharyngeal wall were spared. Partial involvement of the right pyriform sinus was again noted. PET/CT scan demonstrated avidity only within the laryngeal structures without any evidence of nodal involvement or distant disease (Figure 2(b)). The patient subsequently underwent biopsy of the right supraglottic lesion; pathology was consistent with MALT lymphoma (Figure 2(c)).

**Figure 3:**
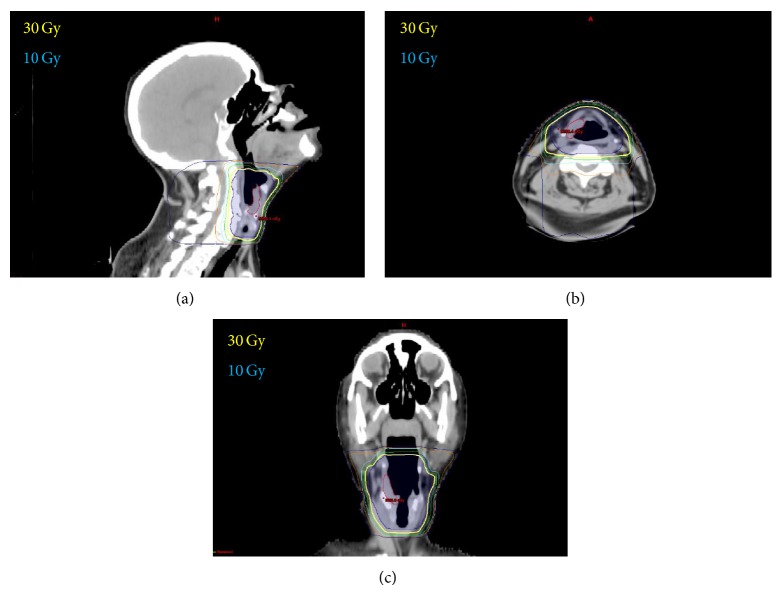
3D conformal radiation therapy of MALT lymphoma of the larynx. Dose distribution in the saggital (a), axial (b), and coronal (c) planes. Yellow line represents the 30 Gy isodose line.

The patient received definitive radiation to 3000 cGy in 15 fractions to the larynx and hypopharynx (Figure 3). The patient tolerated the treatment well developing only mild odynophagia and hyperpigmentation of the skin without any breakdown.

**Figure 4:**
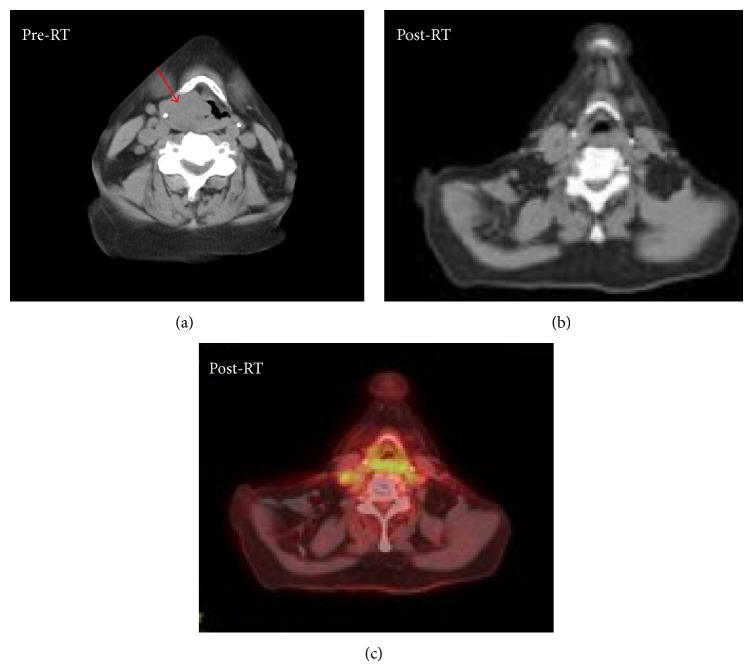
Complete response following radiation therapy. The mass (red arrow) present on CT-based imaging performed at diagnosis (a) has resolved 3 months following radiation (b) and there is no corresponding PET avidity (c).

At a follow-up visit 1 month after completing treatment, the patient's odynophagia has resolved and she is tolerating a full PO diet. She has also had improvements in her voice. PET/CT performed 3 months after completing treatment demonstrated a complete resolution of the tumor subsequently (Figure 4), which was confirmed by fiberoptic examination.

## 3. Discussion

Non-Hodgkin lymphomas (NHL) are tumors of the lymphoid tissues, derived from clonal expansion of  B cells, T cells, natural killer (NK) cells, or their precursors. There are over 12 different types of NHL, including diffuse large B cell (31%), follicular (22%), and MALT lymphoma (5%) [26]. MALT lymphomas are considered extranodal marginal zone B-cell lymphomas under the current world health organization classification system.

MALT lymphomas have been associated with immune system dysregulation from autoimmune disorders like Sjogren's syndrome [27] or sustained immune stimulation from chronic infections with* Chlamydia psittaci*,*  Campylobacter jejuni*, and* Borrelia burgdorferi* [28]. In the stomach, they are most commonly associated with chronic infection with* Helicobacter pylori* [29, 30]. While information on a prognostic parameter such as LDH would be helpful, it was unavailable from an outside institution. Interestingly, our patient had neither a history of autoimmune disease nor an infection with the previously mentioned pathogens.

Radiation therapy represents a definitive treatment option for MALT lymphoma in patients with localized disease. For gastric MALT lymphomas, without t(11;18) status, antibiotics may not have been first line therapy. A radiotherapy regimen of 30 Gy in 20 fractions to the stomach and perigastric nodes has previously been reported with excellent clinical outcomes with high rates of pathological complete responses [31]. In 2013, Zucca et al. presented the ESMO Clinical Practice Guidelines for diagnosis, treatment, and follow-up, reporting excellent disease control with RT alone using moderate-dose involved-field radiotherapy using 24–30 Gy to the stomach and perigastric nodes given in 3 to 4 weeks [32]. In its most recent guidelines released in 2014, the International Lymphoma Radiation Oncology Group reports its consensus on target definition and dose prescriptions for nodal non-Hodgkin lymphoma. There is a move away from previously applied extended-field and involved-field RT (IFRT) techniques that targeted nodal regions toward limiting the RT to smaller volumes based solely on detectable nodal involvement at presentation or involved-site RT (ISRT) as the clinical target volume [33]. Both presentations of this patient were treated using 3D conformal IFRT technique in which the entire organ (larynx or stomach) was treated.

The presentation of laryngeal MALT lymphomas is exceedingly rare, with less than 30 previously reported cases in the literature. Of the previously reported cases, only 11 have involved the supraglottic larynx. These lymphomas are highly radiosensitive. For localized disease, radiotherapy is the most appropriate treatment for larynx preservation. Chemotherapy is reserved for recurrent or disseminated disease [34]. The prognosis is generally favorable with some cases showing no evidence of disease up to 96 months after treatment (Table 1).

The larynx, which has little lymphoid tissue, may be involved secondarily in cases of disseminated malignant lymphoma; therefore clinical staging before and after treatment is necessary [34]. When considering radiation as monotherapy, staging becomes of paramount importance. ^18^F-FDG PET/CT has been used for staging laryngeal MALT lymphomas and has provided a basis for both altering the therapeutic strategy and also evaluating the response to radiotherapy [15]. However, despite being proven valuable in detecting sites of disease in some types of lymphomas, ^18^F-FDG PET/CT is not yet the standard method of staging and cannot substitute bone marrow examination. Biopsies from the ileum could add information as to the extent of this patient's initial disease. It could also explain the fact that the lymphoma which was treated with local therapy relapsed in another extranodal site. The above could be also addressed after the representation and give important information for this case.

In conclusion, laryngeal MALT lymphomas represent rare clinical entities without any clear risk factors. For early stage disease, definitive radiation to a low dose represents an excellent treatment option with limited morbidity, excellent functional outcome, and durable disease control.

## Figures and Tables

**Table 1:** Characteristics of the English-language reported cases of supraglottic laryngeal lymphomas from 1997 to 2014.

Study	Age at presentation	Gender	Symptoms at presentation	Treatment	Disease status	Follow-up (mo)
Zhao et al., 2012 [15]	35	F	Hoarseness	2 cycles CHOP, 27 Gy	NED	3
Markou et al., 2010 [18]	67	M	Hoarseness, stridor, dyspnea	Immediate tracheostomy, CHOP	NED	48
Gonzàlez et al., 2009 [19]	42	F	Cough, dysphonia	RT	NED	96
Arndt et al., 2007 [20]	34	F	Hoarseness	Doxycycline, FCR regimen	NED	6
Fujita et al., 2007 [21]	64	M	Hoarseness	CHOP-R	NED	18
Kania et al., 2005 [22]	46	M	Dysphonia	Co_2_ laser excision, Omeprazole, Amoxicillin, Clarithromycin	NED	24
Caletti et al., 2003 [17]	57	M	Hoarseness	Anti-*H. pylori* therapy	NED	46
Cheng et al., 1999 [23]	58	F	Hoarsness and foreign body sensation	30 Gy	NED	12
de Bree et al., 1998 [24]	36	F	Hoarseness	Debulking and 28 Gy	NED	24
Kato et al., 1997 [25]	54	F	Dysphonia	CHOP and RT, lung: surgery and RT	NED	35

CHOP: cyclophosphamide, hydroxydaunorubicin, vincristine, prednisone.

CHOP-R: cyclophosphamide, hydroxydaunorubicin, vincristine, prednisone, rituximab.

FCR: fludarabine, cyclophosphamide, rituximab.

NED: no evidence of disease.

SOB: shortness of breath.

mo: months.

RT: radiotherapy.

Gy: gray.
